# Primary Urinary Bladder Adenocarcinoma with Signet-Ring Cell Differentiation During Pregnancy Managed by Combined Cesarean Delivery and Radical Cystectomy: A Report of a Rare Case

**DOI:** 10.7759/cureus.114108

**Published:** 2026-08-07

**Authors:** Tariq Abdul Hamid, Joanna Jayakumar, Fadhel Yusuf, Ayman AlYammahi, M. Zaid Jarai, Abdalla Aboelkheir, Afrah Ghayoor, Zenab Yusuf Tambawala, Saba Yehya Alsayari, Abdulmunem M Alsadi, Kais Kotiesh, Yaser Saeedi

**Affiliations:** 1 Urology, Dubai Hospital, Dubai Health, Dubai, ARE; 2 Obstetrics and Gynaecology, Dubai Hospital, Dubai Health, Dubai, ARE

**Keywords:** anterior pelvic exenteration, bladder cancer in young patients, gross hematuria, ileal conduit, immunohistochemistry, non-urothelial bladder cancer, pregnancy cancer, primary urinary bladder adenocarcinoma, radical cystectomy, signet-ring cell carcinoma

## Abstract

Primary urinary bladder adenocarcinoma with signet-ring cell differentiation is an extremely rare and aggressive malignancy. Diagnosis during pregnancy is particularly challenging, as hematuria, anemia, abdominal pain, and pelvic symptoms are often attributed to more common obstetric or gynecologic conditions. The coexistence of pregnancy, obstructive uropathy, and rare bladder histology creates significant diagnostic and therapeutic complexity.

We report a rare case of primary urinary bladder adenocarcinoma with mixed mucinous, intestinal, and signet-ring cell differentiation in a 36-year-old pregnant woman. She initially presented with severe anemia and pelvic bleeding, with early imaging suggesting a possible gynecologic mass. She was later lost to follow-up and re-presented at 24 weeks’ gestation with gross hematuria, renal impairment, and bilateral obstructive uropathy. MRI demonstrated a large circumferential bladder mass with bilateral hydroureteronephrosis and possible extravesical extension. Bilateral nephrostomy tubes were placed, followed by cystoscopy and transurethral biopsy, confirming primary bladder adenocarcinoma with signet-ring differentiation. After multidisciplinary discussion, she underwent cesarean delivery followed by radical cystectomy, anterior pelvic exenteration, and ileal conduit urinary diversion. Final pathology confirmed poorly differentiated signet-ring cell adenocarcinoma with deep perivesical invasion and negative margins. She later developed metastatic recurrence requiring palliative systemic therapy and radiotherapy.

This case highlights the diagnostic difficulty of rare bladder malignancies in pregnancy, which may mimic gynecologic disease and present late with obstructive uropathy and severe anemia. Multidisciplinary management and timely surgical intervention are essential; however, signet-ring-predominant bladder adenocarcinoma may follow an aggressive clinical course despite definitive treatment.

## Introduction

Primary urinary bladder adenocarcinoma is a rare histologic subtype, accounting for only a small fraction of all bladder malignancies, and must be distinguished from secondary involvement by colorectal, gynecologic, or other primary adenocarcinomas [1,2]. Within this group, tumors showing signet-ring cell differentiation are particularly uncommon and are generally associated with infiltrative growth, advanced stage at presentation, and poor oncologic outcomes [1-4]. The diagnosis is often difficult because of significant morphologic overlap with metastatic gastrointestinal adenocarcinoma, making clinicopathologic correlation, endoscopic exclusion of an extravesical primary, and immunohistochemistry essential [3-6].

Bladder cancer during pregnancy is exceptionally rare, and the available literature consists largely of isolated case reports and small reviews [7-9]. As a result, diagnosis may be delayed because hematuria, anemia, abdominal discomfort, and urinary symptoms are initially attributed to more common obstetric, gynecologic, or infectious conditions [7,8]. Imaging is also constrained by fetal considerations, which may further delay timely diagnosis and staging [7,8]. Management requires balancing maternal oncologic urgency against fetal maturity, renal preservation, and operative feasibility, ideally through a multidisciplinary approach involving urology, obstetrics, radiology, pathology, anesthesia, neonatology, and oncology [7-10].

We report a rare case of primary urinary bladder adenocarcinoma with mixed mucinous, intestinal, and signet-ring cell differentiation diagnosed during pregnancy and managed with combined cesarean delivery, radical cystectomy, anterior pelvic exenteration, and ileal conduit diversion.

## Case presentation

A 36-year-old woman, gravida 3 para 2, with a history of two previous normal vaginal deliveries and no known major chronic medical illness, initially presented to the emergency department with abdominal pain, heavy vaginal bleeding, passage of clots, and gross hematuria. Her vital signs were as follows: blood pressure 102/65 mmHg, pulse rate 78 beats/min, respiratory rate 18 breaths/min, and body temperature 37°C. She reported that her menstrual pattern had recently become abnormal, followed by persistent intermittent bleeding, which prompted hospital attendance. On admission, she was found to have profound symptomatic anemia, with a hemoglobin level of 4.6 g/dL (Table 1), and required urgent hemodynamic resuscitation with transfusion of four units of packed red blood cells. She also complained of dysuria, for which she received empirical treatment for presumed urinary tract infection.

**Table 1:** Laboratory investigations upon initial presentation

Laboratory investigations	Laboratory values	Reference range
White blood cell	9.7	3.6 - 11.0 x 10³/uL
Hemoglobin	4.6	12.0 - 15.0 g/dL
C-reactive protein	78	<3 mg/L
Procalcitonin	0.07	<0.05 ng/L
Creatinine	0.62	0.6-1.2 mg/dL

**Figure 1:**
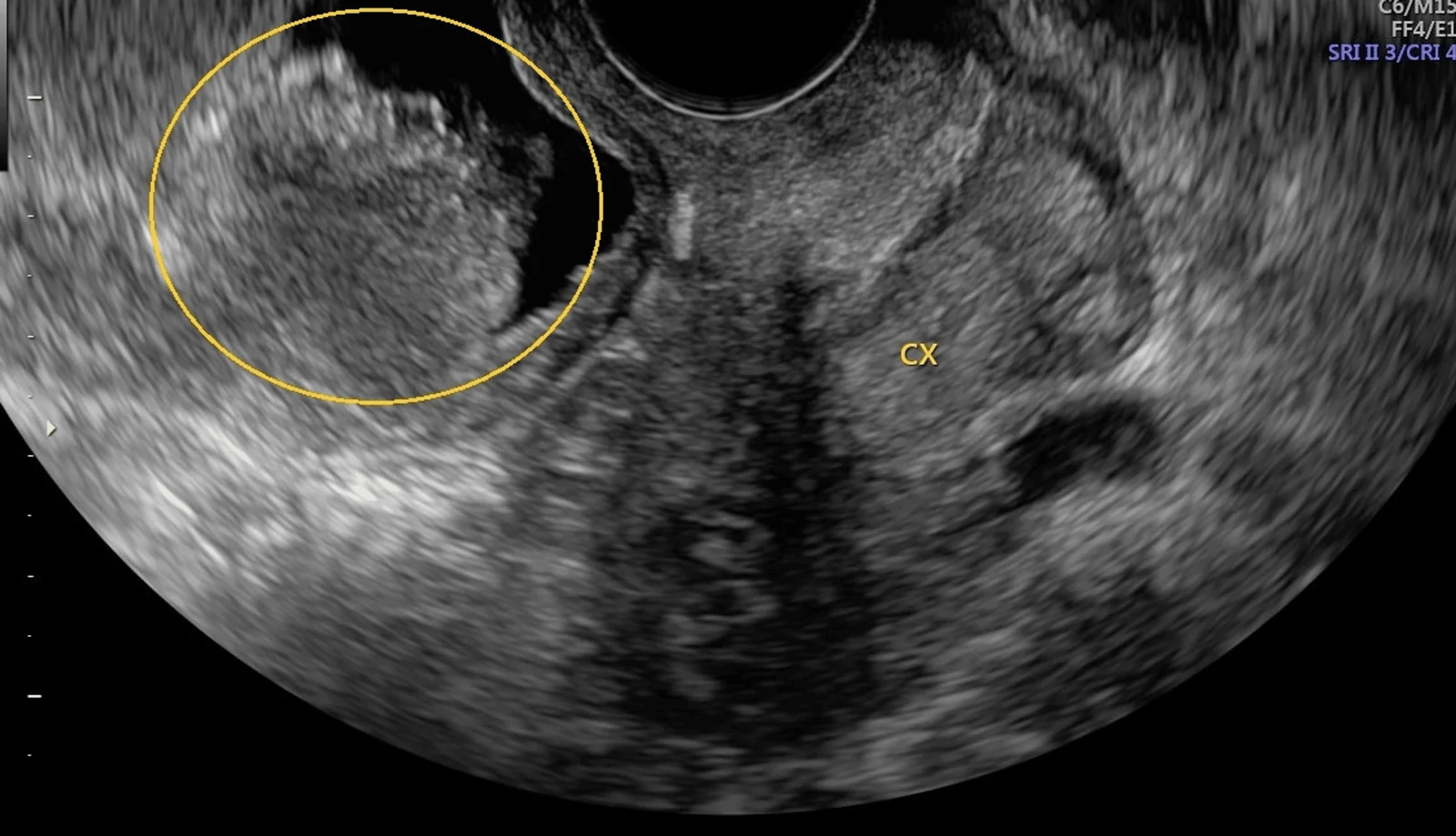
Ultrasound pelvis suggestive of a heterogeneous mass possibly of uterine or adnexal pathology as indicated by the circle.

Initial pelvic ultrasonography identified a large heterogeneous mildly vascular lesion in the anterior pelvis, raising suspicion for uterine or adnexal pathology (Figure 1). The coexistence of visible hematuria prompted further evaluation.

**Figure 2:**
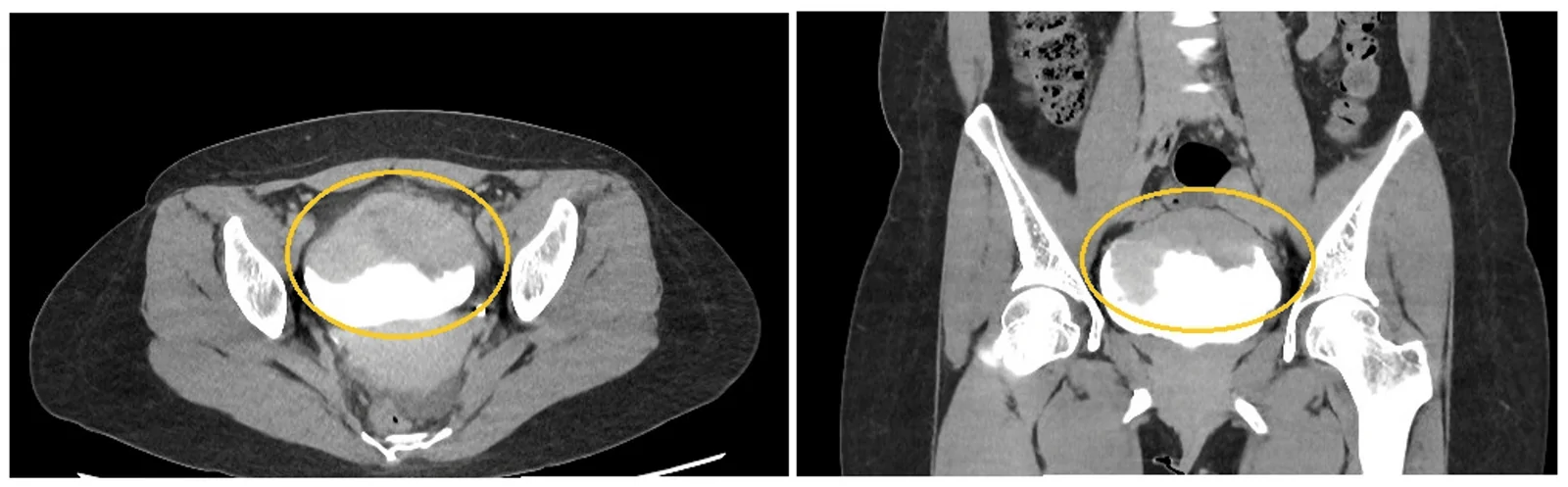
Initial contrast-enhanced CT demonstrating the invasive anterior bladder wall mass. The yellow circle indicates the bladder mass.

A contrast-enhanced CT scan of the chest, abdomen, and pelvis (Figure 2) demonstrated an invasive urinary bladder mass arising from the anterior wall, with flat invasive and papillary-sessile tumoral projections, intramural extension through the muscular layer, and spread into the perivesical fat. No thoracic metastatic disease was identified at that stage. Urologic consultation recommended admission for cystoscopy and transurethral resection of bladder tumour; however, the patient did not complete the planned inpatient evaluation and was subsequently lost to follow-up due to socioeconomic reasons.

Five months later, she re-presented during mid-pregnancy at approximately 24 weeks’ gestation with worsening hematuria, abdominal pain, constipation, anemia, and biochemical evidence of renal impairment. Her vital signs were as follows: blood pressure 124/71 mmHg, pulse 95 beats/min, respiratory rate 16 breaths/min, and temperature of 36.4°C. Obstetric assessment confirmed a viable intrauterine pregnancy. At presentation, her hemoglobin was 6.7 g/dL, and serum creatinine had risen to 4.2 mg/dL (Table 2). A three-way Foley catheter was inserted, and hematuric urine was drained with continuous bladder irrigation. Given concern for advanced local disease in the setting of pregnancy, multidisciplinary evaluation involving urology, obstetrics and gynecology, radiology, anesthesia, and neonatology was initiated.

**Table 2:** Laboratory investigations upon second presentation

Laboratory investigations	Laboratory values	Reference range
White blood cell	10.2	3.6 - 11.0 x 10³/uL
Hemoglobin	6.7	12.0 - 15.0 g/dL
C-reactive protein	138	<3 mg/L
Procalcitonin	0.35	<0.05 ng/L
Creatinine	4.02	0.6-1.2 mg/dL

**Figure 3:**
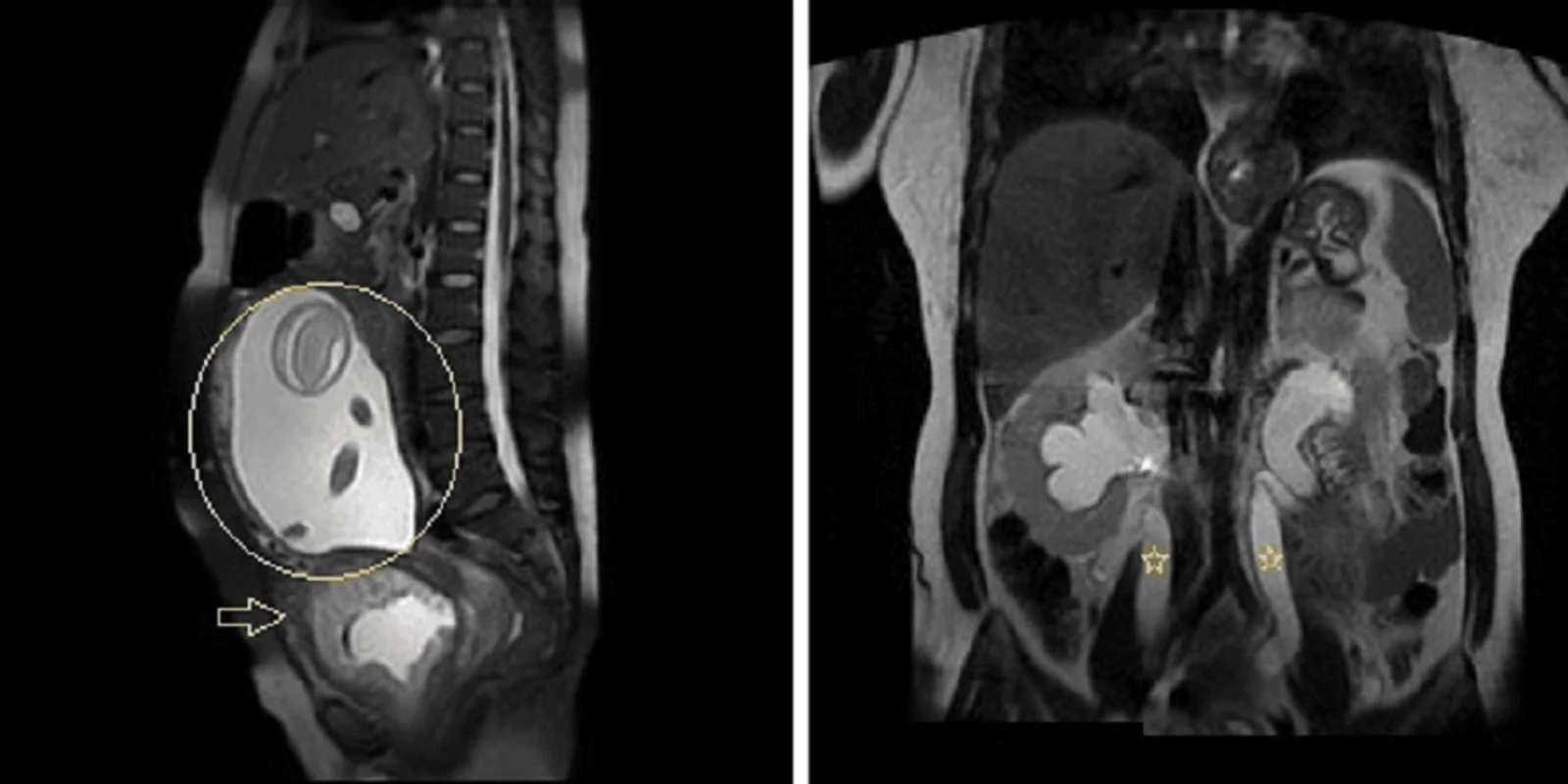
Pelvic MRI during pregnancy showing a circumferential invasive bladder tumor (arrows) with bilateral hydroureteronephrosis (stars) and the adjacent gravid uterus (circle).

MRI of the abdomen and pelvis (Figure 3) demonstrated a large circumferential bladder mass involving the anterior and lateral walls with infiltration of both vesicoureteric junctions, resulting in bilateral hydroureteronephrosis. No definite gross invasion of the lower uterine segment was identified, although mild stranding of adjacent fat planes raised concern for possible extravesical extension. An enlarged internal iliac lymph node was identified. The gravid uterus was displaced by the pelvic mass.

Because the patient had developed obstructive uropathy, bilateral percutaneous nephrostomy tubes were placed, resulting in satisfactory urinary decompression and subsequent normalization of renal function. Once clinically stabilized, she underwent cystoscopy and transurethral resection with deep bladder biopsies. Endoscopically, the tumor was extensive, infiltrating the bladder neck and occupying most of the anterior and lateral walls. Both ureteric orifices could not be identified due to the diffuse nature of the lesion, and only diagnostic debulking was performed.

**Figure 4:**
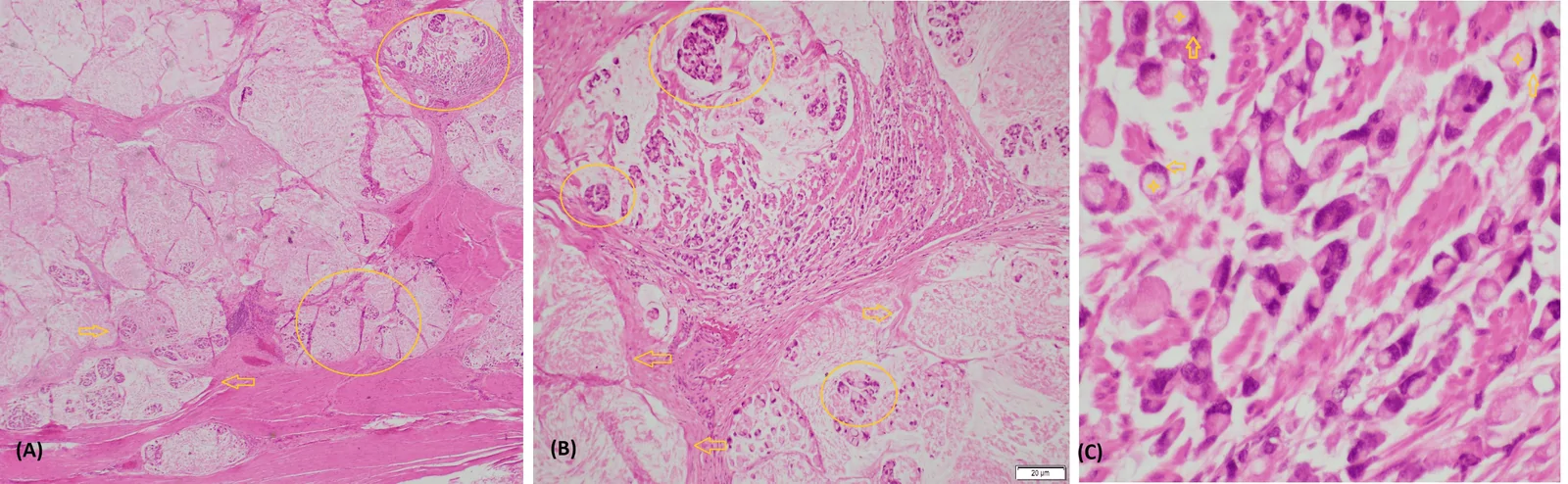
(A) Low-power photomicrograph (×10) showing bladder adenocarcinoma with mucinous areas indicated by the arrow and scattered neoplastic signet-ring cells encircled in yellow. (B) Medium-power photomicrograph (×20) demonstrating a tumor with mucinous areas shown by the arrow and scattered neoplastic signet-ring cells encircled in yellow. (C) High-power photomicrograph (×40) showing neoplastic signet-ring cells with cytoplasmic vacuolization indicated by the asterisk and peripherally displaced hyperchromatic nuclei shown by the arrow.

Histopathological examination of the transurethral resection specimen demonstrated invasive adenocarcinoma with mixed mucinous, intestinal, and signet-ring cell differentiation (Figure 4). In view of the unusual histology and the important differential diagnosis of secondary involvement from a gastrointestinal or gynecologic primary, an extended immunohistochemical panel was performed. The neoplastic cells showed diffuse strong positivity for CK20, CDX2, and GATA3, with membranous beta-catenin staining and no nuclear beta-catenin accumulation. The tumor was negative for ER, PAX8, mammoglobin, TTF1, p16, and the remaining tested markers. Taken together, these findings favored a diagnosis of primary urinary bladder adenocarcinoma of urothelial origin with mixed mucinous, intestinal, and signet-ring cell differentiation, rather than metastatic colorectal or gynecologic adenocarcinoma. To further exclude a secondary gastrointestinal source, upper gastrointestinal endoscopy and colonoscopy were performed and were unremarkable.

The case was reviewed by a multidisciplinary tumor board. Given the patient’s pregnancy, locally advanced bladder tumor, restored renal function after decompression, and the absence of an alternative primary site, definitive surgical management was recommended. Because of the need to balance maternal oncologic urgency with fetal viability, surgery was planned after further fetal maturation following repeated counseling regarding maternal, fetal, and perioperative risks.

**Figure 5:**
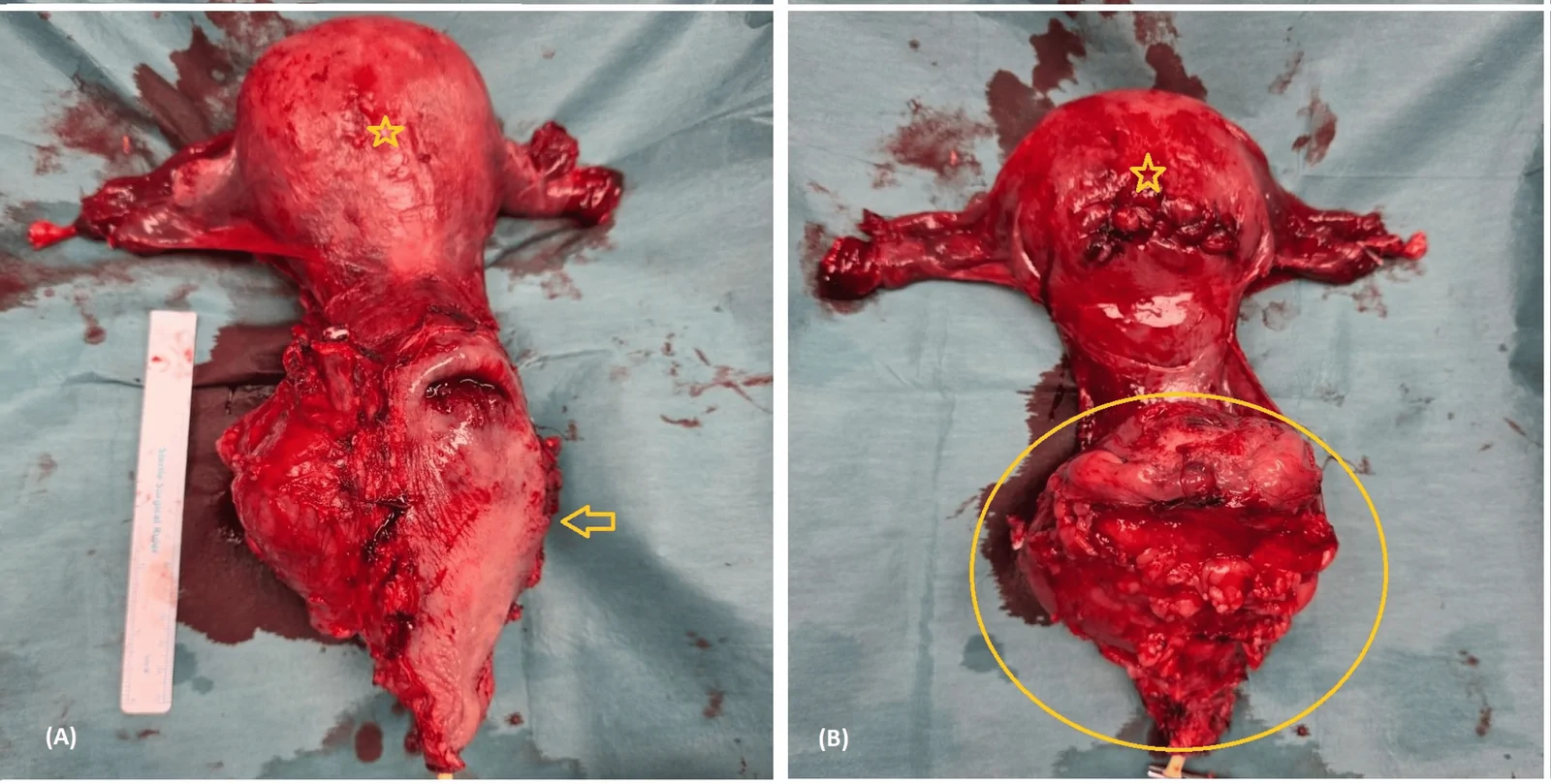
Gross specimen of the resected bladder and adjacent pelvic organs following radical cystectomy and anterior pelvic exenteration. (A) Posterior aspect of the specimen with the arrow showing the anterior vaginal wall and the star representing the uterus in the specimen. (B) Anterior aspect of the specimen with the circle representing the bladder mass and the star indicating the anterior uterus, with the suture line of the performed lower segment cesarean section in the same setting.

She subsequently underwent lower segment cesarean section delivery at 26 weeks gestation, followed immediately by open radical cystectomy, anterior pelvic exenteration, and ileal conduit urinary diversion using the Bricker technique (Figure 5). The postoperative course was uneventful, and she recovered without major immediate surgical complications. A live infant weighing 1.040 kg was delivered with Apgar scores of 3 and 7 at one and five minutes, respectively, and required admission to the neonatal intensive care unit for prematurity, respiratory distress, and hyperbilirubinemia. The neonate was discharged after 56 days in stable condition.

**Figure 6:**
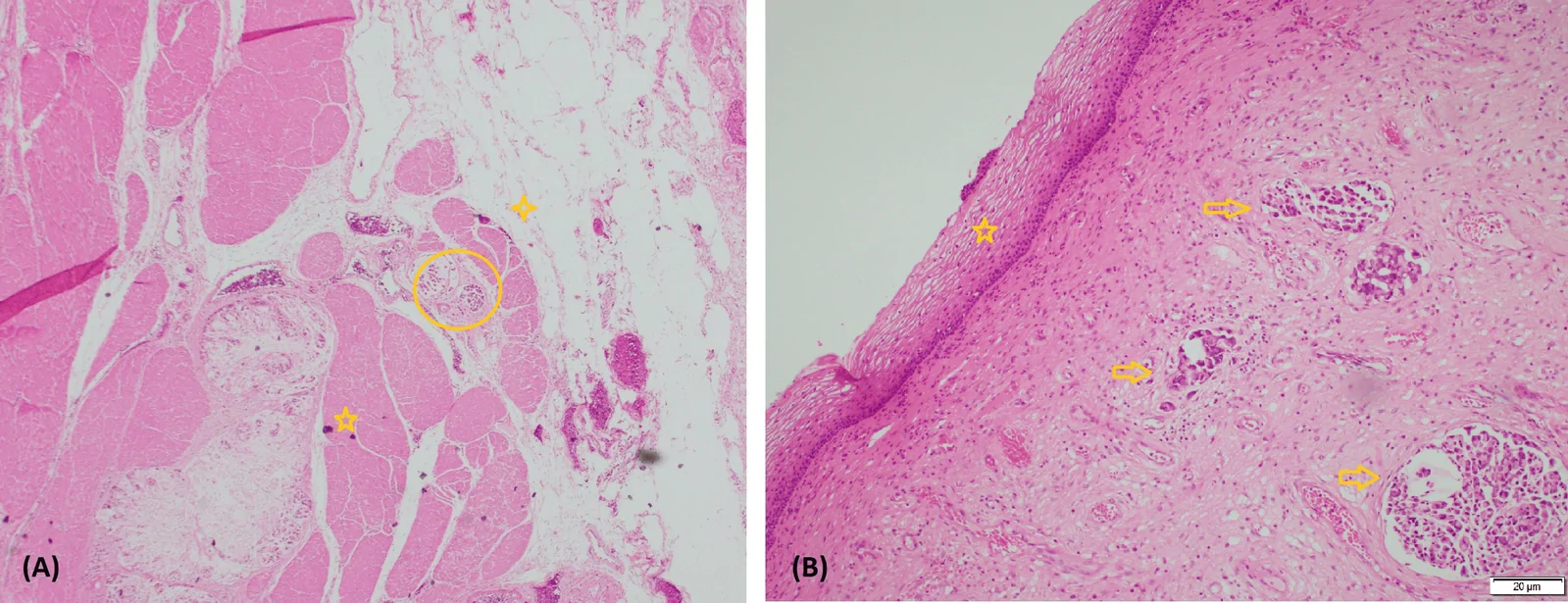
(A) Low-power photomicrograph (×4) showing urinary bladder muscle indicated by the star, with adhesion to the uterus indicated by the asterisk. The yellow circle shows scattered signet-ring cells. (B) High-power photomicrograph (×40) shows cervical squamous epithelium (star) with nests of signet-ring tumor cells indicated by arrows.

**Figure 7:**
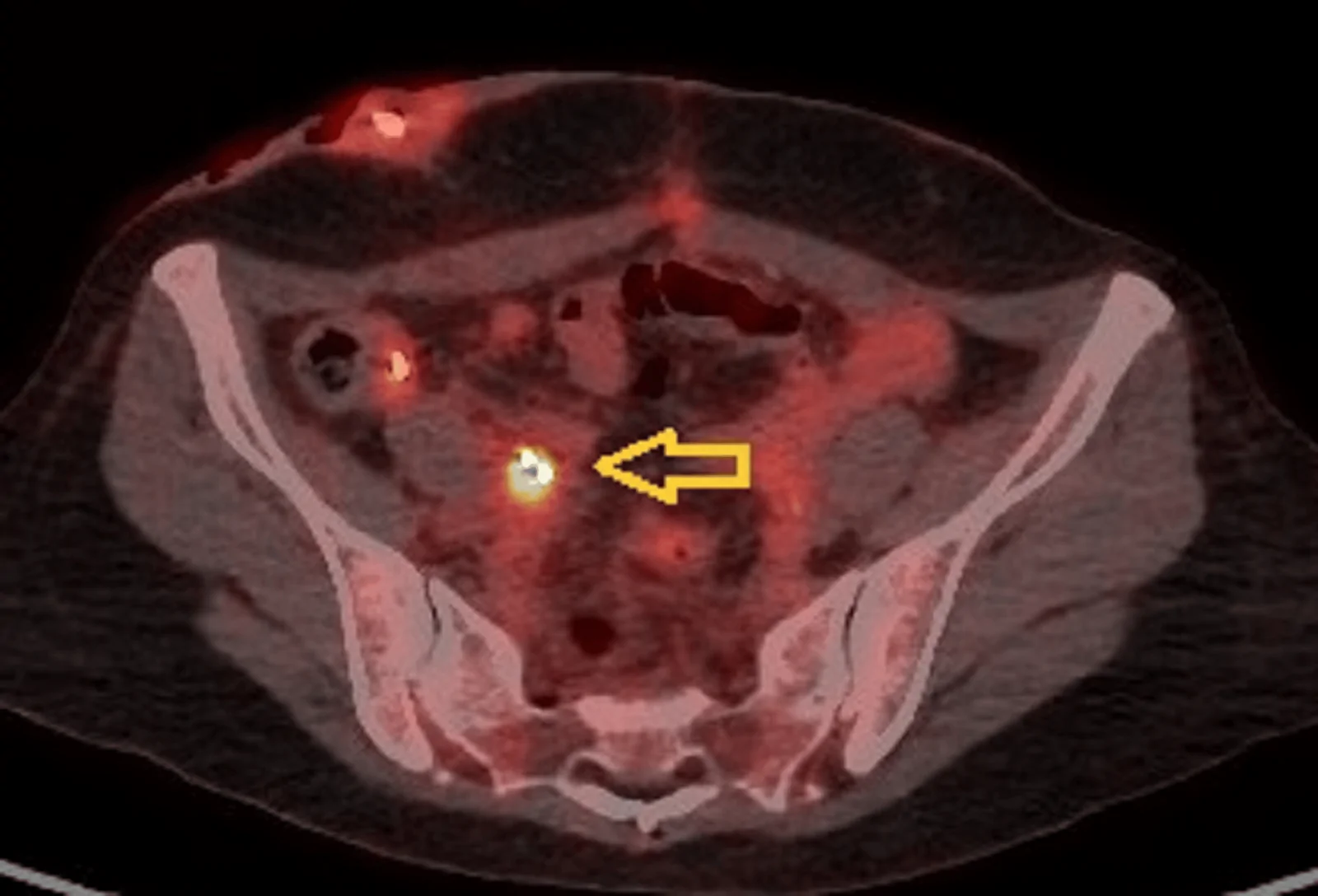
Post-radical cystectomy fluorodeoxyglucose positron emission tomography scan demonstrating metabolically active disease with right iliac nodal uptake (arrow).

Final histopathological analysis of the cystectomy specimen confirmed poorly differentiated signet-ring cell adenocarcinoma of the urinary bladder, staged as pT4a, with invasion into deep perivesical soft tissue and infiltrating the adhesions between the uterus and bladder and the cervical tissue (Figure 6). The right ureteric, left ureteric, and urethral margins were free of invasive tumor, and no lymph nodes were found in the submitted fibro-fatty tissue. A postoperative fluorodeoxyglucose positron emission tomography (FDG-PET) scan demonstrated expected post-surgical changes and a suspicious right iliac nodal focus with mild to moderate metabolic activity but no definite widespread distant metastatic disease at that stage (Figure 7). She was referred to oncology for further assessment and adjuvant treatment planning; however, she did not attend follow-up appointments and was again lost to follow-up due to socioeconomic reasons.

**Figure 8:**
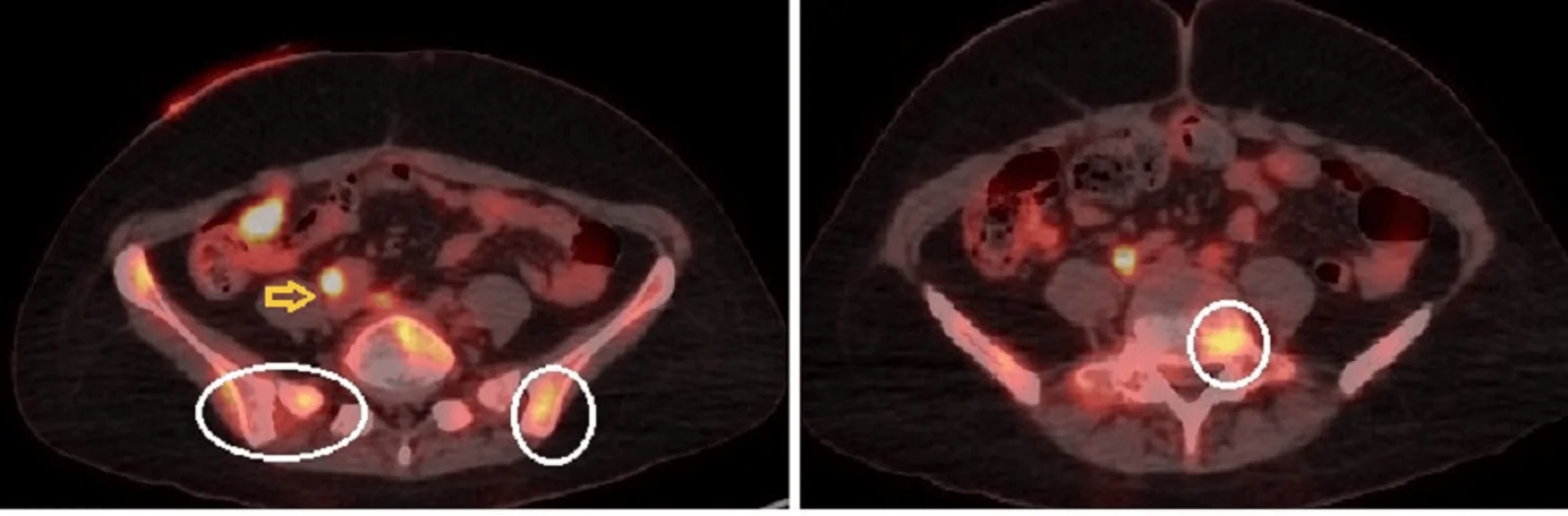
Fluorodeoxyglucose positron emission tomography scan scan status post-radical cystectomy with postoperative changes and an enlarged iliac lymph node with mild hypermetabolism, suspicious of nodal metastasis (arrow). The white circle indicates the focal hypermetabolic lesion in the bone suggestive of skeletal metastasis.

The patient again presented to the emergency department nine months later with urinary tract infection, severe back pain, and progressive weakness of the right lower limb (Table 3). Repeat FDG-PET scan imaging demonstrated enlarged pelvic and retroperitoneal lymph nodes, together with extensive osseous metastatic disease involving the thoracolumbar and lumbosacral spine (Figure 8).

**Table 3:** Laboratory investigations upon third presentation

Laboratory investigations	Laboratory values	Reference range
White blood cell	10.2	3.6 - 11.0 × 10³/uL
Hemoglobin	8.6	12.0 - 15.0 g/dL
C- reactive protein	226	<3 mg/L
Procalcitonin	0.54	<0.05 ng/L
Creatinine	0.73	0.6-1.2 mg/dL

**Figure 9:**
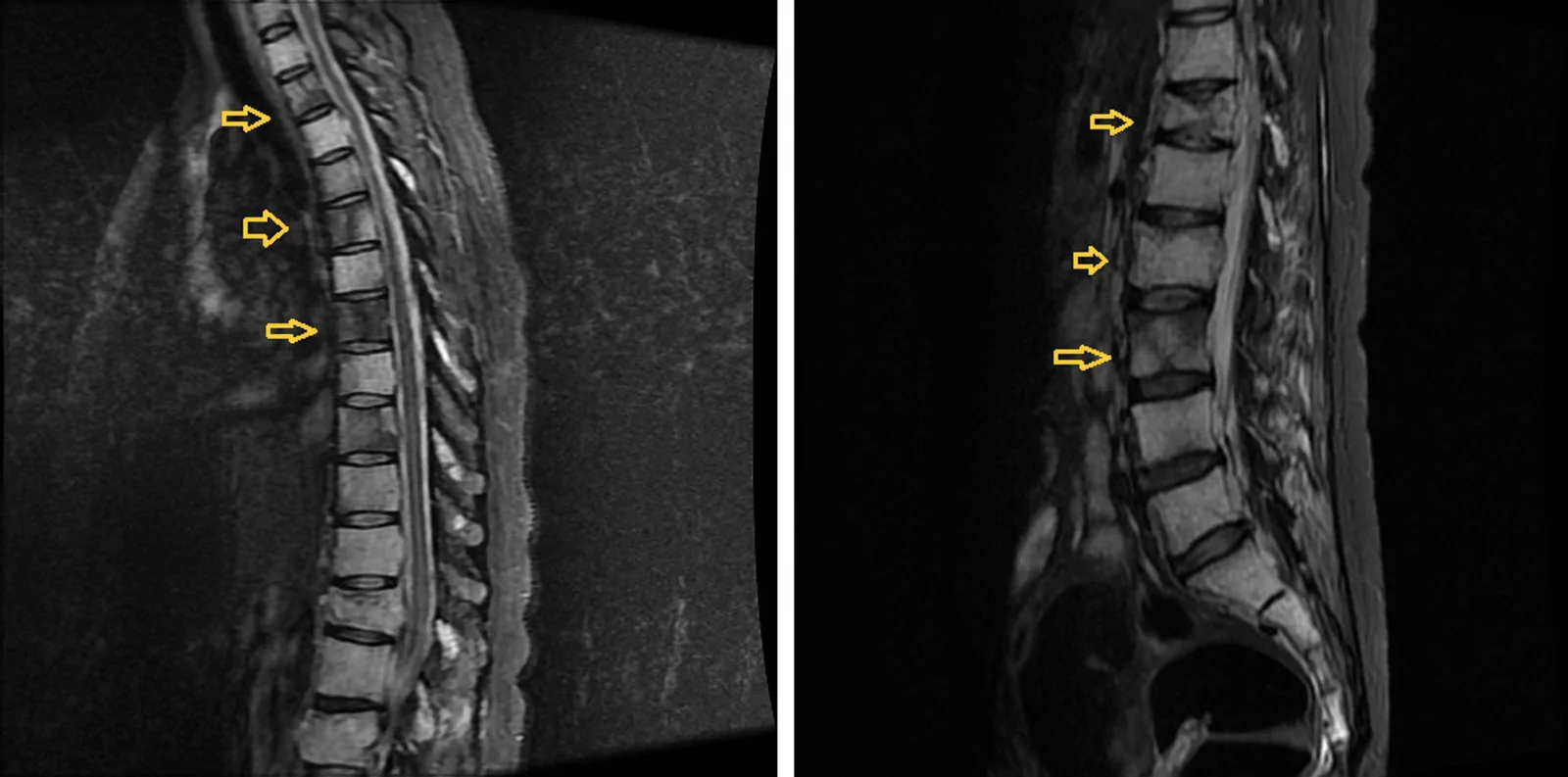
MRI spine suggestive of multiple pathologic compression fractures across the vertebral column due to metastatic involvement (arrows).

Further Imaging with an MRI spine also showed pathological compression fractures and epidural tumor extension, with associated neural element compromise but without overt spinal cord signal abnormality (Figure 9).

**Figure 10:**
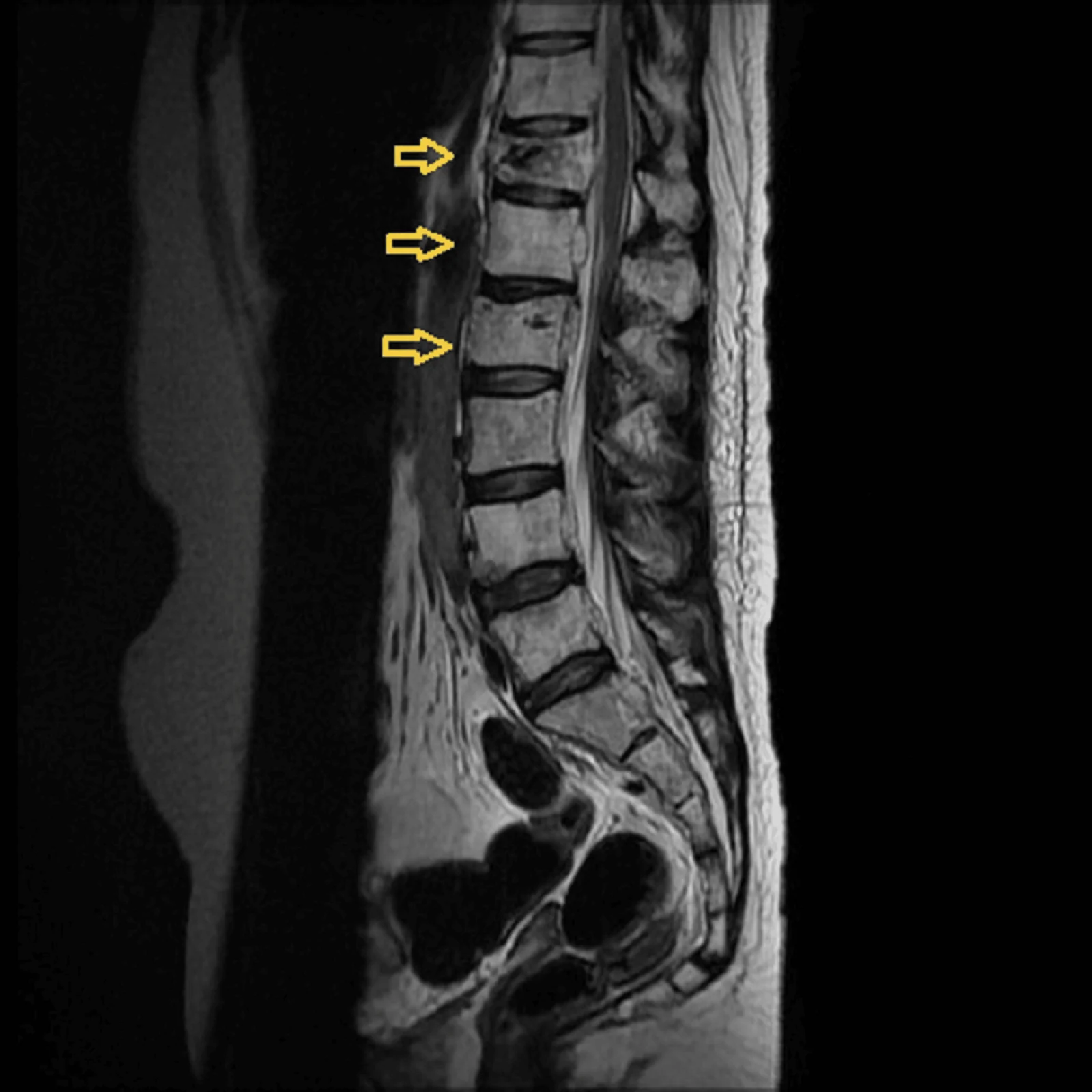
MRI of the spine post kyphoplasty showing a stable lumbar spine, with no retropulsion, facet subluxation, or segmental malalignment (arrows).

After a repeat multidisciplinary review, a palliative strategy was adopted. She underwent vertebral cement augmentation/kyphoplasty for pain control and structural support, followed by port insertion and initiation of first-line palliative chemotherapy with the leucovorin, 5-fluorouracil, and oxaliplatin (FOLFOX) regimen. Follow-up imaging five weeks later after vertebral augmentation/kyphoplasty demonstrated expected post-procedural changes with partial restoration of vertebral body height and cement deposition (Figure 10).

The patient's hospital course was complicated by pain, cytopenias requiring transfusion support, and an intercurrent infection treated with antibiotics. With analgesia, supportive treatment, and systemic therapy, her symptoms improved, and she was discharged with plans for continued chemotherapy, palliative radiotherapy to the spine, and close follow-up with oncology and urology, concluding an otherwise uneventful stabilization course.

## Discussion

This case is notable for the convergence of three uncommon and high-risk features: primary urinary bladder adenocarcinoma with signet-ring cell differentiation and diagnosis during pregnancy.

Primary urinary bladder adenocarcinoma is a rare histologic subtype, and signet-ring cell differentiation represents one of its most aggressive phenotypes [1-4]. These tumors often present at an advanced stage because they grow in an infiltrative pattern, may produce relatively nonspecific urinary symptoms, and are frequently not recognized until hematuria, obstruction, or locoregional invasion becomes clinically evident [1-4,11]. Large contemporary reviews of non-urothelial bladder malignancies similarly emphasize that adenocarcinoma tends to present with biologically advanced disease and poorer survival than conventional urothelial carcinoma, especially when associated with deep invasion or metastatic spread [2,10,11].

Pregnancy added a major diagnostic layer in the present case. Bladder cancer during pregnancy is exceptionally rare, and most published literature consists of isolated case reports and small pooled reviews rather than standardized management algorithms [7-9]. Hematuria in pregnancy may be misattributed to urinary tract infection, stone disease, or obstetric pathology, while severe anemia and pelvic pain may initially direct attention toward gynecologic causes [7,8]. This pattern was clearly reflected here, where the patient initially presented with heavy bleeding and a pelvic mass that appeared possibly gynecologic on ultrasonography, delaying recognition of a bladder primary. MRI proved crucial in defining the true origin of disease, demonstrating circumferential bladder involvement and bilateral obstructive uropathy.

Only a limited number of bladder adenocarcinoma cases during pregnancy have been reported in the literature. Similar reports describe delayed diagnosis due to overlapping obstetric symptoms such as hematuria, anemia, and pelvic pain [12,13]. However, very few cases have documented primary urinary bladder adenocarcinoma with signet-ring cell differentiation complicated by obstructive uropathy requiring nephrostomy drainage and subsequently managed with combined cesarean delivery and radical cystectomy [14]. Recent literature from 2023 to 2025 consists mainly of isolated case reports and small reviews of bladder cancer in pregnancy and non-urothelial bladder malignancies, which consistently highlight delayed diagnosis, advanced stage at presentation, and the rarity of cases requiring coordinated obstetric-urologic oncologic surgery [13].

A second major challenge in this case was pathologic classification. Primary bladder adenocarcinoma, particularly with intestinal or signet-ring morphology, must be distinguished from secondary colorectal adenocarcinoma involving the bladder as well as from gynecologic primaries [1,3-6]. No single immunohistochemical stain is fully definitive; instead, the diagnosis rests on integration of morphology, immunophenotype, clinical findings, and exclusion of another primary site [4-6]. In the present case, diffuse GATA3 positivity supported urothelial lineage, while membranous rather than nuclear beta-catenin staining argued against a colorectal primary [5,6]. The absence of a detectable gastrointestinal primary on endoscopy and the negative gynecologic-associated markers further strengthened the conclusion that this was a primary bladder adenocarcinoma with mixed mucinous, intestinal, and signet-ring differentiation [4-6]. This distinction was clinically essential because it directed management toward definitive extirpative pelvic surgery rather than treatment of a secondary metastatic lesion.

The sequence of management also illustrates the importance of staged prioritization. The patient first required stabilization from severe anemia, then relief of bilateral obstructive uropathy, and only thereafter definitive histologic diagnosis and oncologic planning. Bilateral nephrostomy placement was a key bridging intervention because it reversed renal dysfunction and created a safer window for endoscopic tissue diagnosis and subsequent definitive surgery. For resectable non-urothelial bladder cancers, radical cystectomy with urinary diversion remains the principal curative-intent treatment, although evidence is derived mainly from retrospective series and expert consensus due to the rarity of these tumors [10-15]. In pregnancy, timing must be individualized according to gestational age, disease burden, maternal condition, and fetal considerations [7-10].

In the present case, multidisciplinary coordination allowed temporary stabilization, acquisition of diagnostic tissue, exclusion of a secondary primary, and then definitive surgery timed around fetal viability. The combined lower segment cesarean delivery and radical cystectomy/anterior pelvic exenteration represented a pragmatic solution to competing maternal and fetal priorities in a setting where no high-level guideline can dictate a single ideal pathway.

The final pathology explained the subsequent aggressive course. The presence of pT4a disease with signet-ring cell differentiation and deep extravesical extension is associated with a high risk of recurrence and systemic dissemination [1-4,10,11]. Although postoperative imaging did not initially demonstrate definite widespread distant metastases, later relapse with nodal, osseous, epidural, and probable pleural involvement was consistent with the known biology of signet-ring-predominant bladder adenocarcinoma. The patient’s loss to follow-up further complicated care and limited the opportunity for timely oncologic escalation.

Systemic treatment for metastatic primary bladder adenocarcinoma remains poorly standardized. Unlike urothelial carcinoma, these tumors lack robust prospective trial data, and treatment is often extrapolated from colorectal-type regimens due to enteric differentiation [10,15-17]. Published case-based evidence suggests that fluoropyrimidine- and oxaliplatin-based regimens such as FOLFOX may achieve meaningful responses in selected patients with advanced disease [16,17]. In our case, palliative FOLFOX was therefore chosen once metastatic disease was confirmed. Vertebral cementation, pain control, and palliative radiotherapy further highlight the need for multidisciplinary supportive care beyond systemic therapy alone.

Overall, this case reinforces several practical lessons. Firstly, persistent hematuria in pregnancy warrants structured urologic evaluation and should not be attributed to pregnancy alone. Secondly, primary bladder adenocarcinoma with signet-ring features can mimic gynecologic or gastrointestinal disease both radiologically and histologically. Thirdly, temporary decompression and tissue diagnosis can create the critical bridge to definitive surgery in complex pregnant patients. Finally, even after apparently successful extirpative surgery with negative margins, this histologic subtype may behave aggressively, mandating close surveillance and early oncology engagement.

## Conclusions

Primary bladder adenocarcinoma with signet-ring cell differentiation diagnosed during pregnancy is exceptionally rare and poses major diagnostic and therapeutic challenges. This case demonstrates how severe anemia, hematuria, pelvic imaging ambiguity, and obstructive uropathy can delay recognition of an aggressive bladder primary. Accurate diagnosis required multidisciplinary coordination, cross-sectional imaging suitable for pregnancy, endoscopic tissue sampling, immunohistochemical characterization, and exclusion of a secondary gastrointestinal source. Definitive management with cesarean delivery followed by radical cystectomy and anterior pelvic exenteration was feasible and oncologically justified. Nevertheless, the later metastatic relapse highlights the aggressive natural history of this histologic subtype and the importance of sustained postoperative oncologic follow-up.
